# Commissioning a single photon counting CT simulator for radiotherapy planning: Methods, challenges, and initial results

**DOI:** 10.1002/acm2.70798

**Published:** 2026-09-14

**Authors:** Parminder S. Basran, Devon Richtsmeier

**Affiliations:** ^1^ Department of Clinical Sciences College of Veterinary Medicine, Cornell University Ithaca New York USA; ^2^ Department of Medical Physics BC Cancer ‐ Victoria Victoria British Columbia Canada

**Keywords:** CT scanner, Image quality, Radiation Therapy, Treatment Planning

## Abstract

**Background:**

Photon counting computed tomography (PCCT) offers distinct advantages over standard energy‐integrated detector (EID) CT by offering energy‐resolved measurements, enhanced spatial resolution, and improved soft‐tissue contrast. While PCCT shows great utility in diagnostic imaging, its application as a radiotherapy simulator has not been explored. Commissioning procedures for EID‐based CT simulators are well‐documented, but commissioning a PCCT scanner in a radiotherapy setting has not been investigated.

**Purpose:**

We investigated whether PCCT can be used for radiotherapy simulation by evaluating the geometric accuracy, image quality, dosimetric performance, and relative electron density calibration of a Siemens Naeotom Alpha PCCT scanner with a commercially available carbon‐fiber couch top.

**Methods:**

Commissioning followed AAPM Task Group 66 guidelines, involving electromechanical evaluation, image quality assessment, and radiation safety verification. The CATPHAN 604 phantom was used to evaluate CT number accuracy, uniformity, spatial resolution, low contrast detectability, and geometric accuracy across multiple clinical protocols. Dosimetric measurements were performed using CTDI phantoms with a 100‐mm pencil ion chamber. Relative electron density (RED) calibration was performed using an Advanced Electron Density phantom at selected tube voltages (120 and 140 kVp), slice thicknesses (0.4–10 mm), and reconstruction filters. Statistical analyses included repeated‐measures ANOVA with post‐hoc pairwise comparisons to assess protocol and position effects on Hounsfield unit values.

**Results:**

Geometric accuracy measurements revealed in‐plane spatial linearity of 50.00 ± 0.06 mm (nominal 50 mm). CT number accuracy aligned with manufacturer specifications, except for bone, using specific protocols. The mean discrepancy between measured and nominal CTDI_vol_ was 3.8 ± 4.6%, with excellent agreement on the dose‐length product (0.9 ± 2.3%). Relative electron density measurements suggested statistically significant, but clinically insignificant, variations across protocols (mean HU shifts: ‐23.0 to 7.9 HU). Position‐dependent effects suggested mean HU reductions up to 23.2 HU at peripheral locations. Iterative metal artifact reduction (iMAR) differentiated Titanium and Stainless Steel metallic implants without significantly affecting surrounding tissue CT numbers (*P >* 0.08).

**Conclusions:**

The Siemens Naeotom Alpha PCCT scanner satisfies the requirements noted in **task** group reports for a radiotherapy simulator. The system's enhanced spatial resolution, spectral imaging capabilities, and flexible retrospective reconstruction have the potential to offer advantages for radiation oncology applications, including improved target delineation and treatment planning accuracy.

## 1 INTRODUCTION

Computed tomography (CT) simulators serve as the primary imaging tool for radiotherapy treatment planning, enabling the delineation of target volumes, tissue characterization for dose calculations, and integration with multimodality images, such as magnetic resonance imaging (MRI) and positron emission tomography (PET). More recently, dual‐energy CT technologies have provided novel opportunities in radiation oncology.^1^ Some advantages of using dual‐energy CT as a radiotherapy CT simulator include improved image quality,^2^, ^3^ reduction of metal artifacts,^4^, ^5^, ^6^ and improvements in material characterization for radiation dose.

Photon‐counting CT (PCCT) represents a major recent advance in CT detector technology.^3^, ^7^, ^8^, ^9^ Unlike traditional CT systems that measure the total energy of X‐ray photons within a detector (energy‐integrated detectors, or EIDs), PCCT systems have the ability to count individual photons at the detector and provide energy‐resolved measurements. This spectral capability enables material‐selective imaging, substantial enhancements in soft‐tissue contrast, and significantly reduced beam‐hardening and metal artifacts. Additionally, PCCT systems have substantially higher spatial resolution due to the elimination of interpixel septa and electronic noise, as well as lower radiation dose requirements: these features make PCCT an attractive alternative to EID‐based CT.^2^, ^9^

While PCCT has been explored in the contexts of diagnostic imaging and spectral imaging research, the integration of PCCT as a radiotherapy simulator has yet to be demonstrated. Schwartz *et al.* provides an excellent review of the technology behind PCCTs, along with the advantages and limitations they pose.^9^ Accurate tissue characterization and artifact‐free imaging are particularly significant for radiotherapy, where even minor inaccuracies in contouring or dose calculation can directly affect the success of treatment. PCCT may provide enhanced material differentiation, which can improve delineation of tumor and critical structures and render other possibilities, such as virtual non‐contrast imaging, material decomposition, and render sub‐millimeter targeting technically feasible.

Commissioning a CT simulator for radiotherapy requires rigorous evaluation to ensure its accuracy, precision, and compatibility with clinical oncology workflows. Procedures for commissioning CT systems, CT simulators, and diagnostic dual‐energy CT scanners have been well‐established and widely adopted; however, the same cannot be said for PCCT. The purpose of this work is to address this knowledge gap by outlining and reporting on the tests for commissioning a PCCT in our clinical facility.

## 2 METHODS

### 2.1 System description

A Siemens Naeotom Alpha PCCT equipped with dual X‐ray tubes operating at 90, 120, and 140 kVp was commissioned for radiotherapy simulation. The system features an 82 cm bore and cadmium telluride detectors that directly convert photons to electronic signals, eliminating the scintillation step required in EIDs. Spatial resolution reaches 0.16 mm × 0.11 mm × 0.11 mm with reconstruction matrices up to 1024×1024 pixels and slice thicknesses ranging from 0.2 to 10 mm. The extended Hounsfield unit scale spans ‐8,192 to +57,343 HU (2^16‐bit precision), substantially exceeding the ± 3071 HU range of EID‐based CT scanners.

A convenient feature of this system is its ability to retrospectively specify reconstruction parameters, including slice thickness, field of view, and kernels, from raw projection data. This eliminates the need for repeated patient scans when imaging parameters require adjustment. Full spectral capabilities, including virtual non‐contrast imaging and material decomposition, require acquisition at 140 kVp. The system was configured with a Q‐Fix carbon fiber flat‐top couch that emulates the Varian Exact couch used on our institution's linear accelerator, and installed three translatable radiation therapy patient‐alignment lasers positioned 50 cm from the isocenter for patient positioning.

### 2.2 Commissioning protocol

The commissioning was broken into three broad categories: electromechanical evaluation, image quality evaluation, and radiation and patient safety, as per AAPM Task Group Reports 66, 233, 291, and the CPQR Report, with considerations documented in AAPM Task Group Reports 233 and 291.^10^, ^11^, ^12^, ^13^ Details for measurement parameters (Supplementary Table S1), tabularized results, and figures are provided in the supplementary documentation. A condensed version of the protocols examined in this study is shown in Table 1. Gantry tilt tests were not performed during this commissioning because they are not used in our radiotherapy procedures. Couch deflection under load was also evaluated.

**TABLE 1:** CT protocols evaluated for baseline image quality.

Protocol Name	kVp	mAs	Pitch	Slice Thickness [mm]	Filters
Head and Neck^+^	140	83, 159^*^, 239	0.35	0.4, 1.0^*^, 2.0	Hr44^*^, Hr76, Qr40
Head and Neck^+^	120	218	0.35	0.4, 1.0^*^, 2.0	Hr44^*^, Hr76, Qr44
Thorax	140	127^*^, 159	1.0	0.4	Br44, Br76^*^, Bl64, Qr44
Non‐Contrast Brain	140	231, 233	0.35	0.4	Hr44, Hr76, Qr40
Forelimb	140	41	0.8	0.4	Br44, Br76, Qr40
CTA Thorax‐ Protocol A	140	9	1.5	0.4	Br44, Br76, Qr40
Abdomen	140	10	0.8	0.4	Br44, Br76, Qr40
Limb	250	88, 159	0.35, 0.80, 1.00	0.4	Br44, Bl64, Br76
Trunk	140	11	0.8	0.4	Br44, Br76, Bl64, Qr40
Trunk	120	6	0.85	0.4	Br44, Br76, Bl64, Qr40
Pelvis	140	10	0.8	0.4	Br44, Br76, Qr40
CTA Thorax—Protocol B	140	9	1.5	0.4	Br44, Br44, Br64
Venous Contrast	140	9	1.5	0.4	Br44. Br64, Qr40
Delayed Contrast	140	9	1.5	0.4	Br44. Br64, Qr40
Delayed	140	9	1.5	0.4	Br64

The Qr filters were reconstructed with a monoenergetic energy of 70 keV.

The protocols marked with asterisks

(*) identify the reference scan conditions for comparative RED and image‐quality analysis. Protocols with hatch

(+) identify protocols assessed with phantoms shifted from the image center. CTA protocols differ by filters.

**FIGURE 1:**
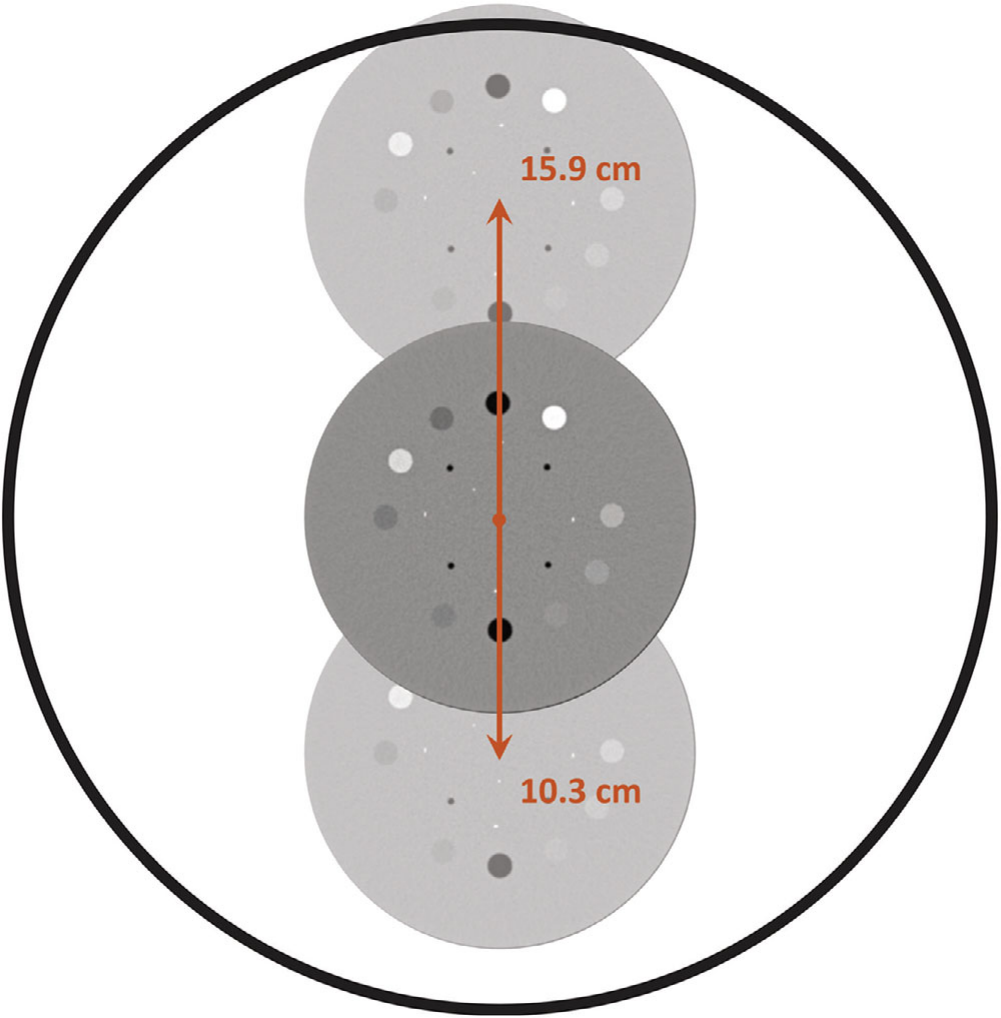
Positions of the Catphan 604 phantom relative to the CT isocenter.

The CATPHAN 604 phantom was scanned using a variety of clinical protocols (see Supplementary Table S2 and Supplementary Figure S1), and the images were analyzed with Pylinac open‐source software.^14^ Evaluation included CT number accuracy for nine materials (air at ‐1000 HU to Teflon at ∼1000 HU), uniformity measured as the standard deviation among five regions of interest, and spatial resolution quantified through the relative modulation transfer function (rMTF) from line‐pair patterns. Low contrast detectability using the Rose criterion (visibility threshold = 0.15) was applied to phantom rods ranging from 2–15 mm in diameter with contrast levels of 0.3–1.0%. Geometric accuracy was verified through slice‐thickness measurements using wire ramps and spatial linearity using 50 mm‐spaced phantom holes (see Supplementary Table S3). Image quality metrics were also extracted with the phantom in different positions inside the bore (Figure 1).

CTDI_100_ was measured using a 100‐mm pencil ion chamber in three nested cylindrical phantoms (32, 16, and 10 cm diameter, Sun Nuclear, Melbourne, FL) at the center and four peripheral positions (Unidose Tango, 30009, PTW, NJ). The CTDI_w_, CTDI_vol_, and dose‐length product values were computed and compared against scanner‐reported values (See Supplementary Table S4 for measurement details).

### 2.3 Relative electron density measurements

The Advanced Relative Electron Density (RED) Phantom (Sun Nuclear, FL) was scanned using clinically relevant protocols that varied kVp (120, 140 kVp), slice thickness (0.4, 1, 2 mm), pitch (0.35–1.00), and mAs (83‐218), with multiple reconstruction kernels (see Supplementary Table S5). These protocols represent the range of scanning conditions used across different anatomical sites in our radiotherapy practice. Custom MATLAB scripts extracted mean CT numbers from each material insert, and repeated‐measures ANOVA was performed, along with paired t‐tests and Wilcoxon signed‐rank tests, to identify protocol‐dependent variations, with Holm‐corrected pairwise comparisons when statistical significance was detected. When significance was observed, Cohen's d was computed for effect size (0.2, 0.5, 0.8, > 1.2, representing thresholds for small, medium, large, and very large effects, respectively).

**FIGURE 2:**
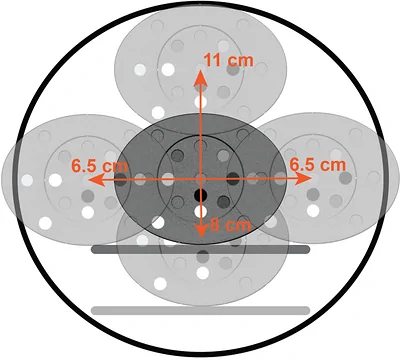
Positions of the RED phantom relative to the CT isocenter. Note that while it is technically feasible to image the RED phantom lower than 8 cm if using the curved couch, the 50 cm couch top does not permit the patient anatomy to be imaged below this vertical couch position. Scans were acquired after the phantom was horizontally leveled and aligned with the axes of the central imaging plane.

Image quality has been shown to vary within the field of view –particularly for large‐bore CT simulators– which can influence estimates of electron density.^15^ HU accuracy was evaluated by scanning the RED phantom at multiple positions within the 80 cm extended field of view, including vertical couch adjustments and lateral positioning (Figure 2). This assessment used both 120 and 140 kVp acquisitions at three slice thicknesses to identify position‐dependent HU shifts relative to the isocenter.

Metallic artifacts may also significantly bias the CT numbers in CT scans. Two water‐equivalent phantom inserts with clinically used pedicle screws (titanium alloy (Ti‐6Al), stainless steel) were scanned to evaluate metal artifact performance. Scans were acquired at 120 and 140 kVp, both with and without iterative metal artifact reduction (iMAR), and mean CT numbers were quantified for the metal implants and surrounding materials to assess artifact suppression and the system's ability to differentiate between high‐Z materials.

## 3 RESULTS

### 3.1 Image quality

Measured CT numbers for materials in the CATPHAN phantom aligned well with manufacturer specifications across most protocols. The only notable deviation occurred for 50% bone at ∼725 HU, where 140 kVp measurements showed substantially higher variability. This discrepancy appeared consistently across all CT angiography protocols (CTA, Delayed, and Venous protocols in Supplementary Table S2). Higher kernel levels produced better spatial resolution, as expected. The relationship between kernel sharpness and rMTF values at 50%, 30%, and 10% followed predictable patterns across all protocols tested (see Supplementary Figure S2). Maximum standard deviation among the five uniformity ROIs reached 3.7 HU, though all but five protocols remained well within clinical requirements (< 1.5 HU) for radiotherapy CT simulation. Protocols using our standard 0.4 mm slice thickness detected a few low‐contrast rods. Detection improved markedly with thicker slices, demonstrating the expected trade‐off between spatial resolution and contrast sensitivity. In‐plane spatial linearity measurements averaged 50.00 ± 0.06 mm compared to the expected 50 mm distance. Slice thickness measurements showed consistent underestimation. Analysis of protocols acquired at a nominal thickness of 0.4 mm revealed measured values scattered when using a 5‐slice average but condensed around ∼0.29 mm when using a 9‐slice average (Supplemental Figure S3): this is approximately 0.11 mm below the nominal value. Varying slice thickness from 0.2 to 6.0 mm affected image quality as expected (Supplemental Figure S4 & S5). Spatial resolution remained essentially constant across this range, likely because the 2 mm‐thick bar patterns remained clearly visible at all tested thicknesses. Low contrast detectability increased with thicker slices, while both uniformity variation and image noise decreased, as expected.

### 3.2 Position‐dependent image quality

The CATPHAN phantom position within the field of view had measurable effects on image quality. Quantitative metrics of overall uniformity as a function of phantom position are shown in Table 2. Moving the phantom vertically by 26.2 cm (from lower to upper positions) produced substantial changes in image quality parameters.

**TABLE 2:** Uniformity, background noise, and CT number accuracy as a function of the CATPHAN phantom position.

Metric	Baseline (Center position)	Lower Position	Upper Position
Uniformity (max. difference between center and peripheral ROI)	1.45 HU	1.49 HU	5.38 HU
Noise (std. dev. in the low contrast module background)	16.4 HU	18.5 HU	25.9 HU
CT Number Accuracy	106.4 HU	106.1 HU	107.2 HU

The phantom center in the upper position was 15.9 cm above the center in the middle position, while the center in the lower position was shifted 10.3 cm down. The data shown here is for the Br44 kernel images.

The 12 o'clock ROI in the upper position, being furthest from isocenter, drove the substantial increase in uniformity variation from 1.45 HU to 5.38 HU.

Spatial resolution (rMTF) decreased with off‐center positioning, showing steeper degradation for the lower position at higher spatial frequencies (Supplementary Figure S6). This asymmetry likely reflects the spatial distribution of the high‐resolution bar patterns within the phantom. The largest patterns sit below the phantom center, placing them relatively further from isocenter in the lower position despite less vertical displacement.

### 3.3 Radiation dosimetry

Measured CTDI_vol_ values differed from nominal by 3.8% ± 4.6% (mean ± SD), while DLP measurements differed by 0.9% ± 2.3% (Supplemental Table S‐3). Agreement was excellent overall. The largest discrepancy appeared in the ChildAbdomen protocol at 90 kVp, where measured CTDI_vol_ exceeded nominal by 0.4 mGy (10.1%) and DLP by 1.3 mGy (5.6%).

### 3.4 Relative electron density: protocol dependence

Mean CT numbers across all RED phantom inserts for 120 and 140 kVp protocols showed no statistically significant difference (*P =* 0.108), though visual separation appeared at higher density inserts (Supplemental Figure S7). Comparing individual 120 kVp protocols against baseline revealed statistically significant differences for two protocols (*P <* 0.01), with mean HU shifts ranging from ‐5.1 to +7.9 HU. More protocols showed statistical significance at 140 kVp (nine total; *P <* 0.01), with mean HU shifts spanning ‐23.0 to +7.8 HU. While statistically significant, these shifts remain within clinically acceptable ranges for radiotherapy dose calculation: typically ± 20 HU corresponds to < 1% dose calculation error for most tissue types.

### 3.5 Relative electron density: position dependence

Position‐dependent HU variations emerged as the most clinically relevant finding. Moving the RED phantom away from isocenter produced a mean HU reduction of 23.2 HU, with individual measurements showing reductions up to 63.5 HU at 95% confidence intervals (Table 3). Effect sizes (Cohen's d) for position‐dependent HU variations ranged from 0.05–0.26 for soft tissue‐equivalent materials (near water density) to 2.87–3.74 for cortical bone, indicating that practical significance scales with material density deviation from water. High‐density materials (CaCO_3_ 50%, cortical bone) and low‐density materials (lung LN‐300) showed large to very large effect sizes (*d =* 0.70–3.74), while materials near water density demonstrated negligible position dependence (*d <* 0.3).

**TABLE 3:** Results for relative electron density measurements as distance from the image center was increased.

				Confidence Interval	
kVP	mAs	Slice Thickness [mm]	Mean Difference [HU]	5%	95%
120^*^	218	0.4	−15.7	−24.5	−6.9
120	218	1.0	−21.7	−31.9	−11.5
120^*^	218	2.0	−23.7	−44.6	−2.8
140	127	0.4	−24.5	−56.2	7.3
140	127	1.0	−24.5	−56.2	7.3
140	127	2.0	−29.3	−59.0	0.4

The reference conditions are scans with asterisks in Supplementary Table S5. Protocols with asterisks (*) are those protocols whose RED measured data are statistically different from baselines when the RED phantom is positioned away from the central axis (*P <* 0.04).

**FIGURE 3:**
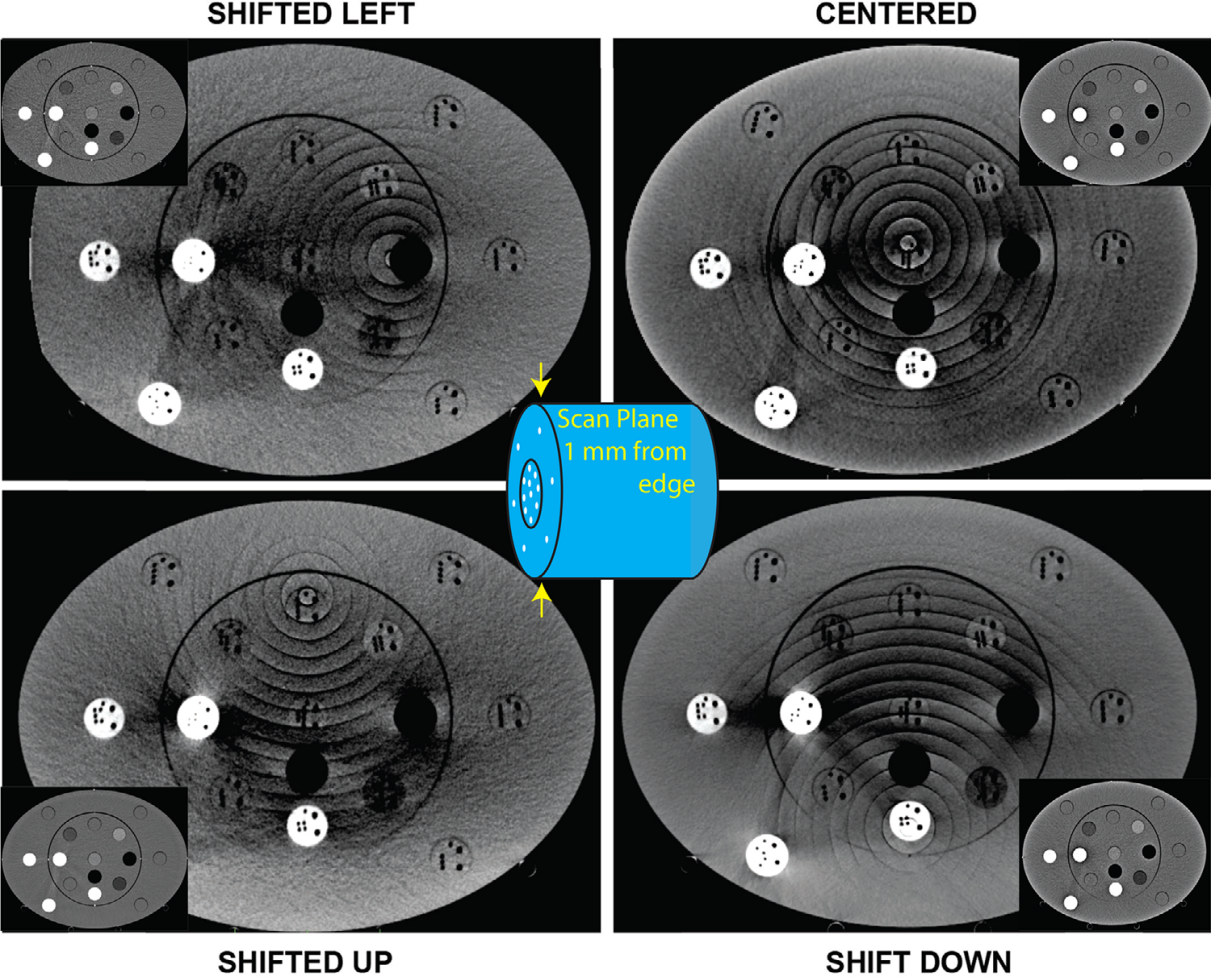
CT images illustrating extreme discontinuity in the RED phantom (superiorly) and the SPCCT ring artifact when the phantom is positioned in various locations in the CT bore. All images were obtained with 0.4 mm slice thickness, 140 kVp, Hr44. The window = 400, level = 40 for all images. The inset images display the phantom at the middle of the phantom, where these artifacts are negligible.

This systematic peripheral HU reduction appears as a ring artifact in the reconstructed images. An extreme example is highlighted in Figure 3, which displays sharp discontinuities between the phantom and rods at the boundary of the RED phantom. A distinct ring artifact at the imaging center is clearly apparent when there are extremely sharp discontinuities in the z‐axis of the scan plane.

### 3.6 Metal artifact reduction performance

**FIGURE 4:**
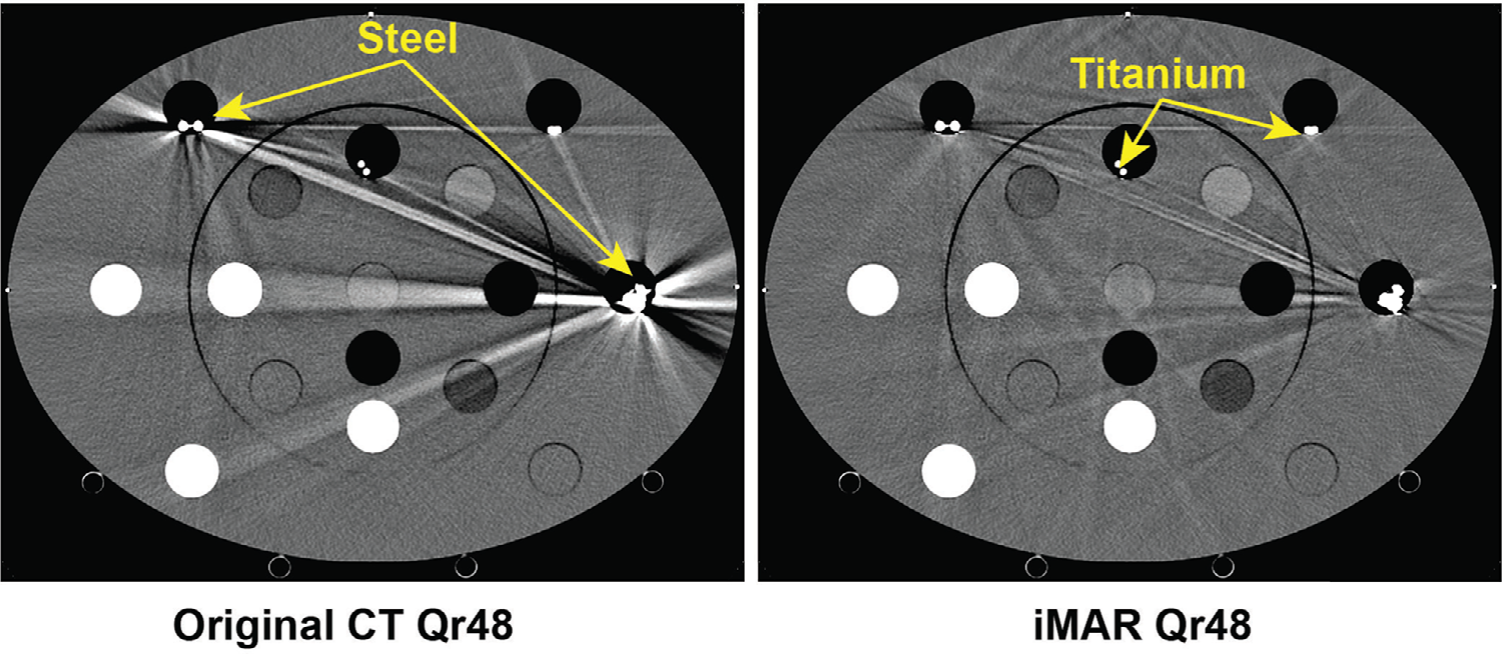
An example of an Original CT (no metallic artifact correction) and iterative metallic artifact reduction (iMAR) image obtained with 140 kVp, 0.6 mm slice thickness. Spinal fixation (both steel and titanium) hardware was inserted into one of the vials typically containing water.

**FIGURE 5:**
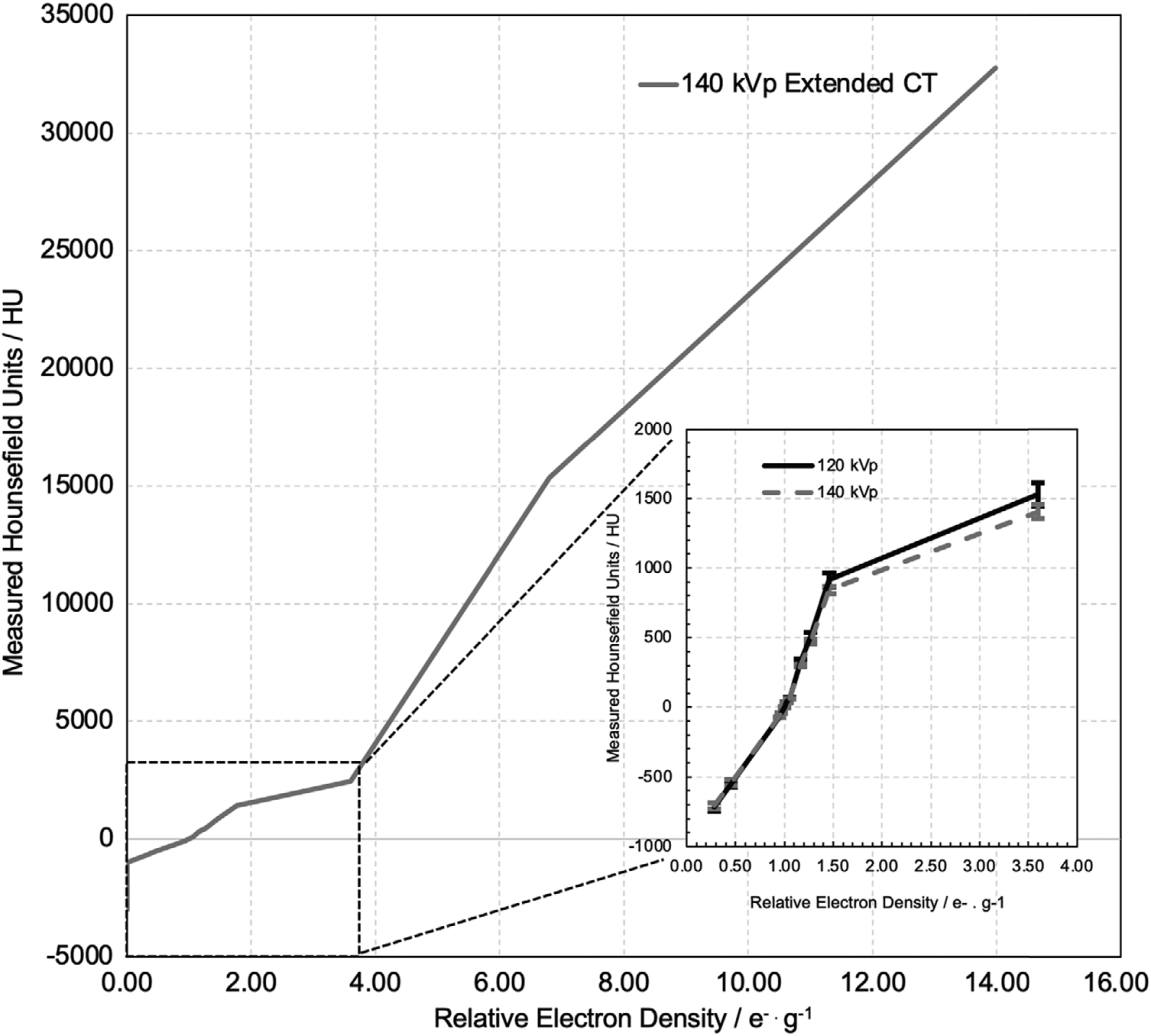
Averaged and extended Relative Electron Density curves for 140 kVp using measured data when including measurements from titanium and stainless steel. Note the extended range in measured HU units (‐3000 to 32766 HU) within the treatment planning system (Varian, Eclipse, V18), although measured CT numbers have been observed to exceed 32766 HU (maximum bit resolution = 216, or 65536 Hounsefield Units).

Stainless steel pedicle screws measured 32,509 ± 3,310 HU across all protocols (average, standard deviation), while titanium alloy (Ti‐6Al) screws measured 5,329 ± 1,018 HU (Figure 4). This ∼6‐fold difference remained consistent across all scanning protocols and reconstruction parameters tested. Paired t‐tests comparing iMAR‐corrected scans against (no metallic artifact corrected) CT scans showed no significant differences in surrounding tissue CT numbers (*P >* 0.08 for all protocols). Mean CT numbers for water‐equivalent vials adjacent to metal implants remained within ± 3 HU of baseline measurements after iMAR application. Figure 5 shows the extended electron‐density calibration curve, including metal inserts. CT numbers for the stainless steel implants exceeded 32,767 HU in all protocols, requiring the PCCT's 2^16‐bit HU scale (‐8,192 to +57,343 HU) for accurate representation. Metal artifact reduction performance remained consistent across kVp settings (120 and 140), slice thicknesses (0.4, 1, 2 mm), and reconstruction filters tested.

## 4 DISCUSSION

Geometric accuracy met clinical standards, with in‐plane measurements within 0.1 mm of expected values. Slice thickness measurements consistently underestimated nominal values by ∼0.1 mm but remained within TG‐233 tolerances (± 0.5 mm for < 1 mm nominal thickness). Baseline image quality metrics fell within manufacturer specifications and established tolerances from TG‐66, TG‐233, and CPQR guidelines.^10^, ^11^, ^12^ Measured radiation doses agreed well with nominal values (mean difference 3.8% for CTDI_vol_), with typical radiotherapy simulation doses of 5–20 mGy at 140 kVp. These values are consistent with published values for EID‐based CT simulators.^16^, ^17^

The observed peripheral HU reductions (mean 23.2 HU, maximum 63.5 HU) represent a previously undocumented characteristic of PCCT in radiotherapy. While statistically significant, these variations have minimal clinical impact on dose calculations. Thomas demonstrated that a 10% change in electron density produces dose variations of only 0.6‐1.4% for photon therapy, indicating that the ± 20‐30 HU variations observed in this study (corresponding to approximately ± 2‐3% density changes) would produce dose errors well under 1%.^18^, ^19^ Proton therapy range calculations demonstrate greater sensitivity to CT number variations, with Schaffner and Pedroni reporting that conversion uncertainties of ± 1.1‐1.8% in tissue composition correspond to range uncertainties of 1–3 mm, yet remain clinically acceptable within standard treatment planning margins.^20^ The 23.2 HU mean peripheral reduction observed in this study falls within these thresholds. However, the magnitude exceeds typical cupping artifacts reported for modern EID CT simulators, which generally show < 10 HU variation across similar field sizes. Effect size analysis (Cohen's d) demonstrated that position‐dependent HU variations scale with material density deviation from water, with negligible effects for clinically relevant soft tissue materials, compared to large effects for extreme‐density materials such as cortical bone, confirming minimal clinical impact for routine radiotherapy applications. The asymmetric pattern (greater degradation in upper vs. lower phantom positions despite different distances from isocenter) suggests detector geometry or reconstruction algorithm contributions rather than simple beam‐hardening effects common in EID systems.

Our observed 23 HU peripheral reduction exceeds typical cupping artifacts in modern EID CT simulators (generally < 10 HU across similar fields of view). However, the ring artifact pattern (Figure 3) differs from traditional beam‐hardening cupping, suggesting photon‐counting detector‐specific contributions requiring different QA approaches than EID‐based systems. The ring artifact appears most prominently at sharp material transitions and varies day‐to‐day in magnitude, necessitating daily quality assurance with uniform phantom scans. It should be noted, however, that such extreme boundaries along the z‐axis are not common when scanning patients. Initial clinical experience is that the magnitude of these ring artifacts are not as severe, and there is no need for density overrides in patient volumes when these artifacts are present in radiation oncology CT simulations. As a consequence of these artifacts, our center has implemented an additional quality control CT scan of the daily calibration phantom provided by the manufacturer to monitor the extent of this artifact, assessed through visual inspection and careful evaluation of kernels that might enhance these artifacts in routine clinical use. Future work focuses on the long‐term stability of the imaging performance.

The 2^16‐bit HU scale (‐8,192 to +57,343 HU) accommodates extreme values from metal implants that exceed common CT limits, though this requires treatment planning system updates. The ability to differentiate titanium (5,329 HU) from stainless steel (32,509 HU) represents a practical advantage over EID CT systems, where both materials often saturate detector response. The iMAR algorithm reduced streak artifacts without significantly altering surrounding tissue CT numbers (*P >* 0.08) near the metal implants. This contrasts with some metal artifact reduction approaches that introduce distortions in tissue values. The clear differentiation between titanium and stainless steel (a 6‐fold HU difference) offers an advantage over EID systems, where both materials typically saturate.^21^ For the two high‐density materials tested, our results suggest that stainless steel and titanium may be reasonably discriminated and hence modeled in our treatment planning system. True material decomposition for other (high) atomic‐number elements is future work for consideration. The iMAR algorithm maintained surrounding tissue CT numbers within ± 3 HU, which is comparable to or better than published EID metal artifact reduction and post‐processing methods.^6^, ^22^, ^23^, ^24^ The measured CT numbers differ from simulation‐based estimates in published literature, but absolute values matter less than establishing material differentiation. The 6‐fold HU difference enables correct material assignment in treatment planning systems. Validation of geometric fidelity for high‐density objects remains a subject for future work.

Treatment planning systems require extension beyond the traditional ± 3071 HU range to accommodate the PCCT's 2^16‐bit scale (Figure 5). Implementing this extended range involves several practical considerations, including database modifications, extending CT‐to‐density calibration curves beyond traditional ranges, revalidating clinical workflows to ensure proper handling of extreme values, and potential regulatory review depending on institutional requirements. While high‐density objects are rarely imaged, reconstructed voxels occasionally exceed 32,767 HU due to reconstruction artifacts. These occurrences are infrequent and predominantly noise‐related, making their dosimetric impact negligible for routine planning.

Scan protocol changes produced statistically significant HU shifts (up to 23 HU at 140 kVp), but these remain within clinically acceptable ranges. Greater variability at 120 kVp suggests standardizing on 140 kVp protocols. This approach also enables full spectral reconstruction capabilities (virtual non‐contrast, material decomposition) from archived data. The retrospective reconstruction capability reduces repeat scans due to incorrect initial parameters, potentially lowering cumulative patient dose.

PCCT offers advantages in material differentiation and spatial resolution over EID systems, though clinical benefits for radiotherapy require further investigation. The institution has adopted 1 mm isotropic resolution (140 kVp, 512 × 512 matrix, ∼0.98 mm pixels) for routine CT simulation, though this produces substantially larger datasets. The ability to retrospectively adjust reconstruction parameters has proven valuable for addressing slice thickness, field of view, and artifact reduction needs without additional scanning. The increased spatial resolution and reconstruction flexibility offer opportunities for enhanced target delineation and high‐precision radiotherapy, warranting continued investigation of PCCT applications in radiation oncology.

## 5 CONCLUSIONS

This work describes the commissioning of a new photon‐counting CT for radiotherapy treatment simulation. Using procedures described in professional CT and radiotherapy reports, our findings suggest that, when coupled with a radiation therapy couch and laser alignment systems, the Siemens Naeotom Alpha CT scanner may be safely used for radiotherapy CT simulation. PCCT technology offers a tremendous range of versatility and functionality that has yet to be explored in radiation oncology.

## AUTHOR CONTRIBUTIONS

Both authors contributed equally to this work.

## CONFLICT OF INTEREST STATEMENT

The authors declare no conflicts of interest.

## Supporting information



Supporting Information

Supporting Information

## Data Availability

Data may be made available upon reasonable request.
