# Supplementary material for: Commissioning a single photon counting CT simulator for radiotherapy planning: Methods, challenges, and initial results
Source: J Appl Clin Med Phys. 2026 Sep 14;27(9):e70798. doi: 10.1002/acm2.70798 (PMC13575489; doi:10.1002/acm2.70798)
Supplement: Supplementary file 1 [file ACM2-27-e70798-s001.pdf]

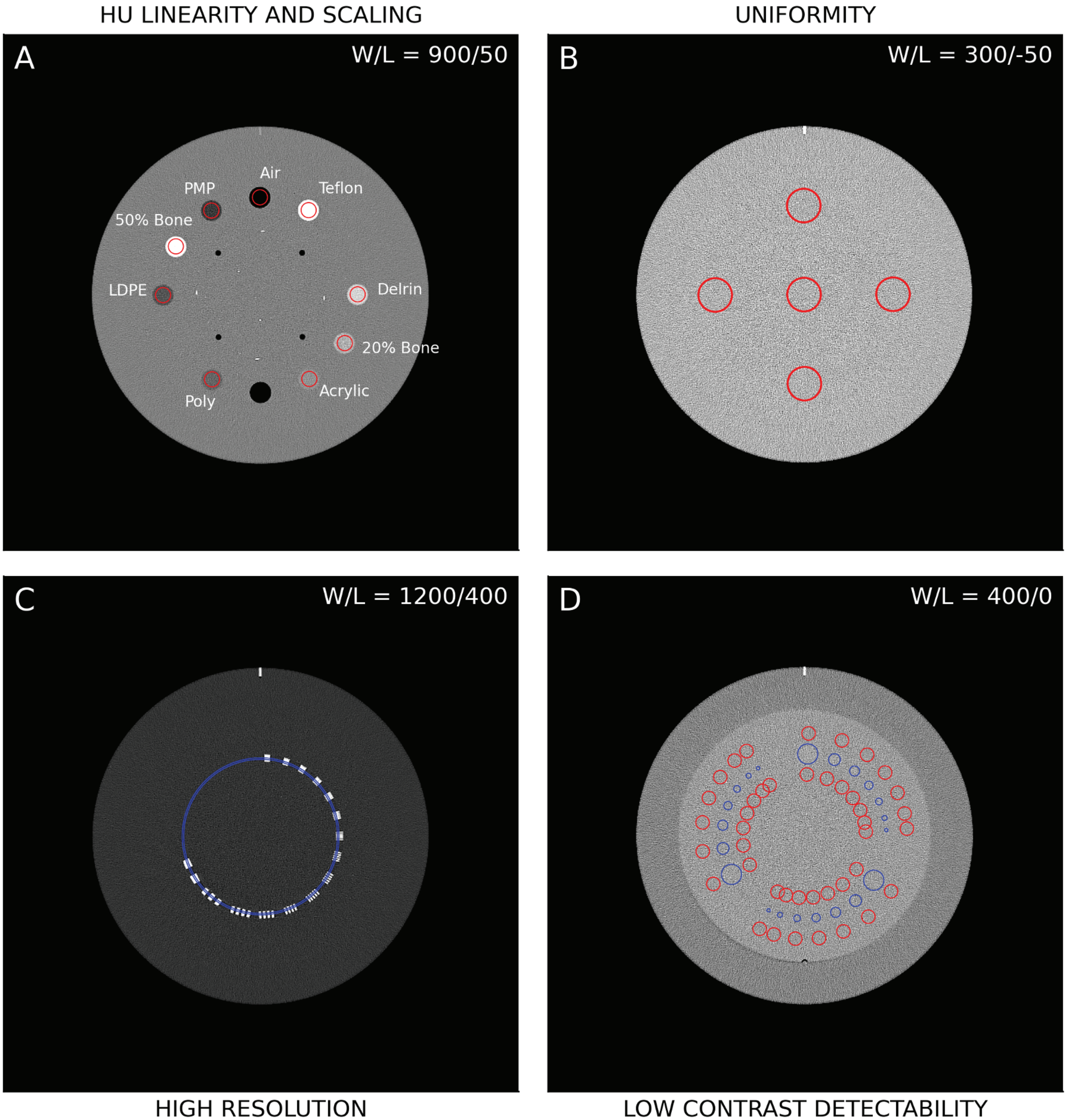

Figure S1: CT images of the CATPHAN 604 phantom with ROIs drawn for the locations where image quality was assessed. (A) The HU linearity and scaling module. ROIs where the mean CT number was evaluated for the various materials are shown in red. (B) The uniformity module. ROIs for the five areas in which uniformity and noise were evaluated are shown in red. (C) The high spatial resolution module. Line profiles were drawn between the two blue lines to evaluate the relative MTF. (D) The low contrast detectability module. ROIs in blue show the locations of the low contrast rods, and ROIs in red show the area where the background CT number was evaluated.

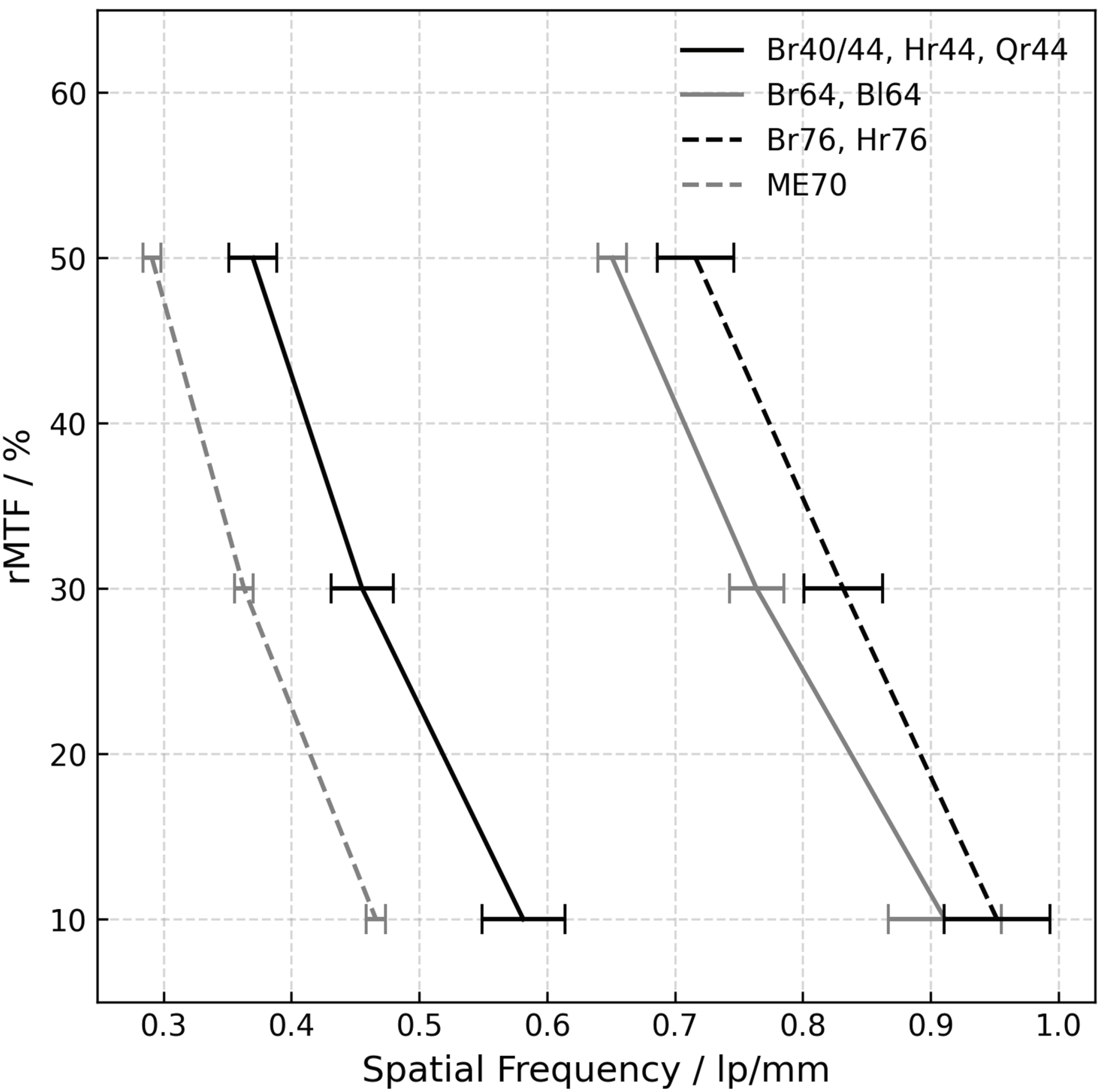

Figure S2: Baseline high spatial resolution relative MTF (rMTF) values. Overall mean 50%, 30%, and 10% rMTF values were calculated over the protocols in Table 2, separated by kernel level, as shown in the legend. Errorbars are the standard deviation over the same protocols.

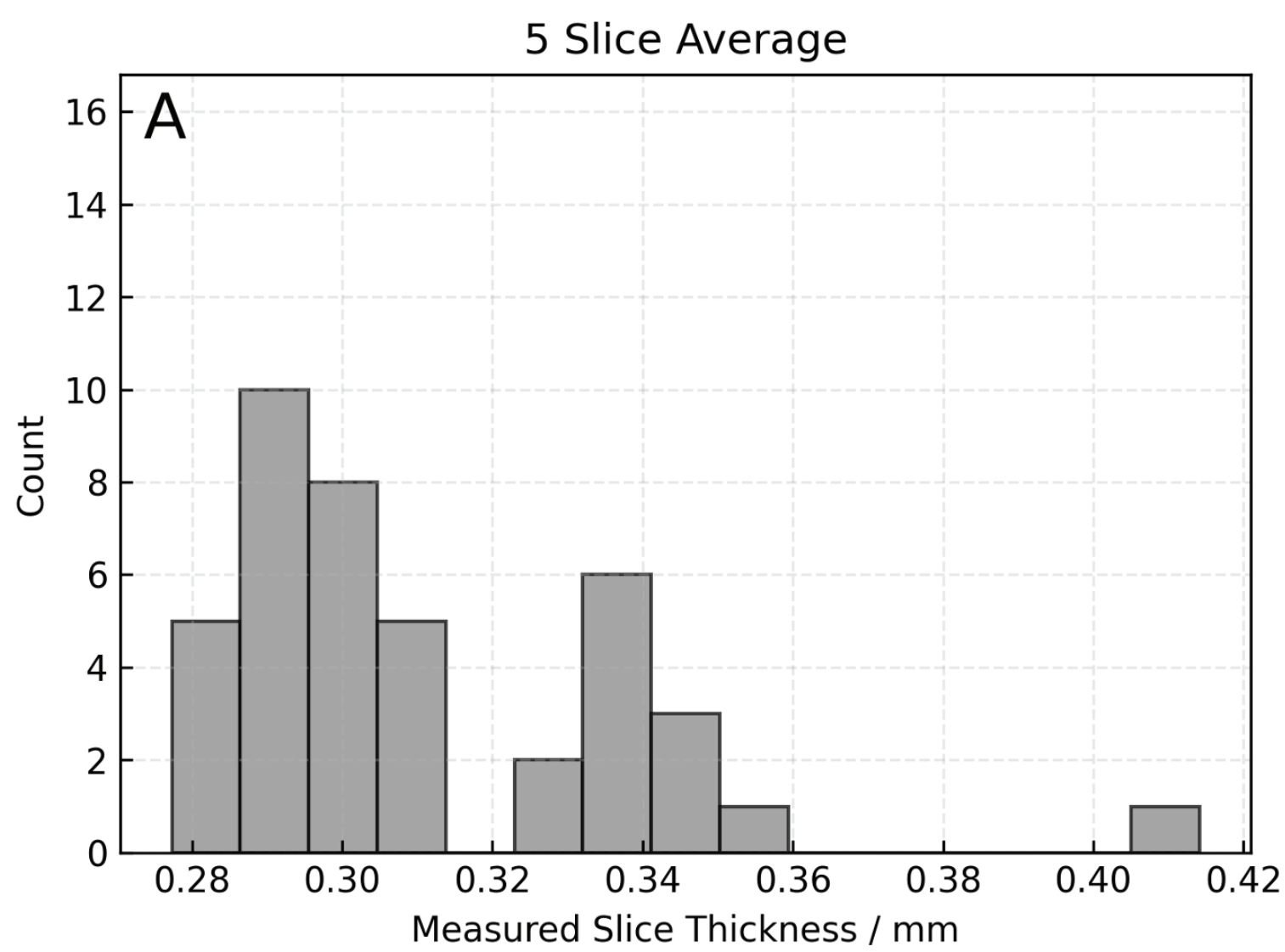

Figure S3: Histograms of the measured slice thickness for all the protocols in Table 2 with a slice thickness of 0.4 mm. (A) Five slices were used in the calculation of the slice thickness. (B) Nine slices were used in the calculation of the slice thickness.

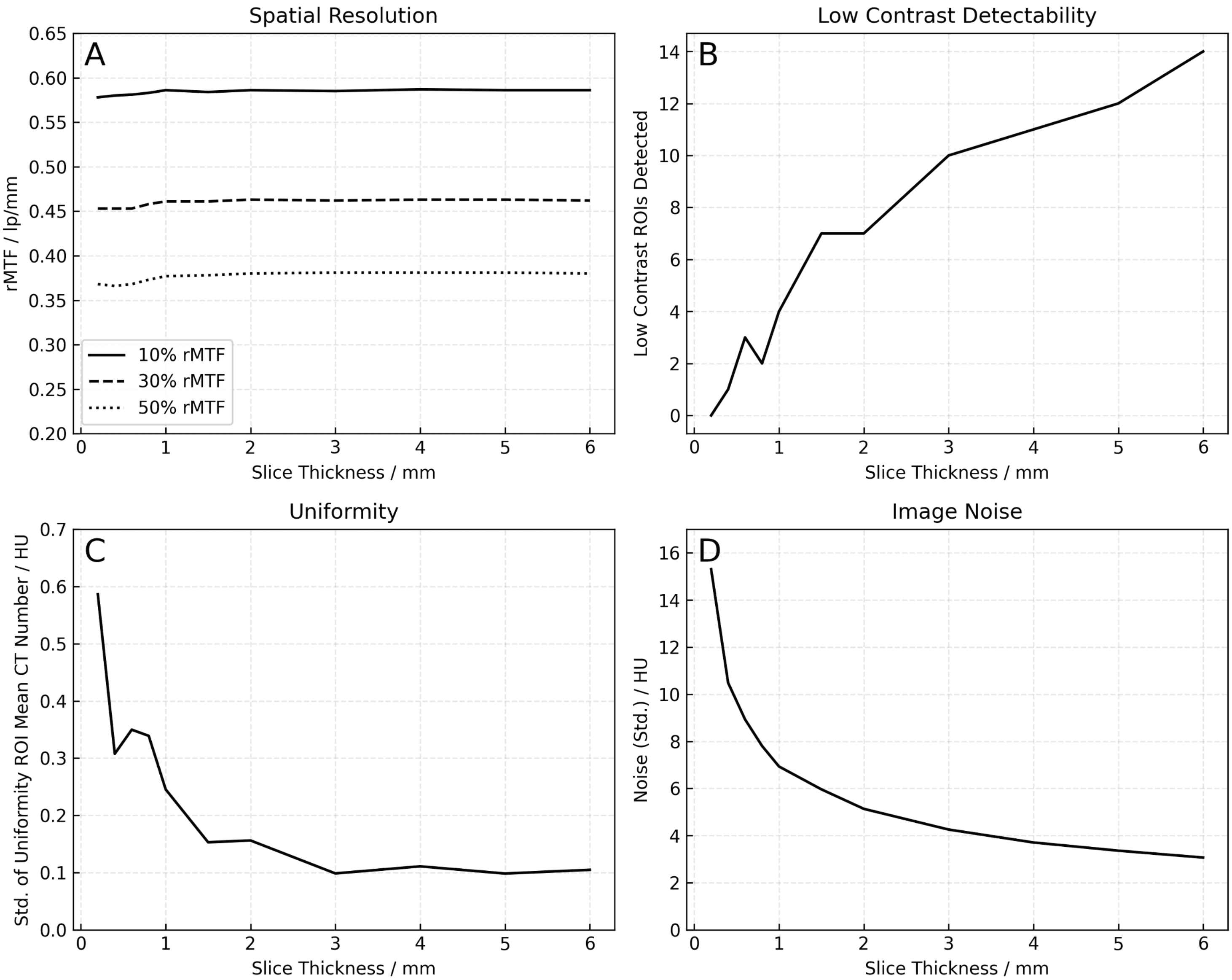

Figure S4: Changes to image quality metrics as a function of slice thickness. (A) The 50%, 30%, and 10% rMTF values. (B) The number of low contrast ROIs detected. (C) The standard deviation between mean CT number in the five uniformity ROIs. (D) Noise as measured by the standard deviation of CT number in the background of the low contrast module.

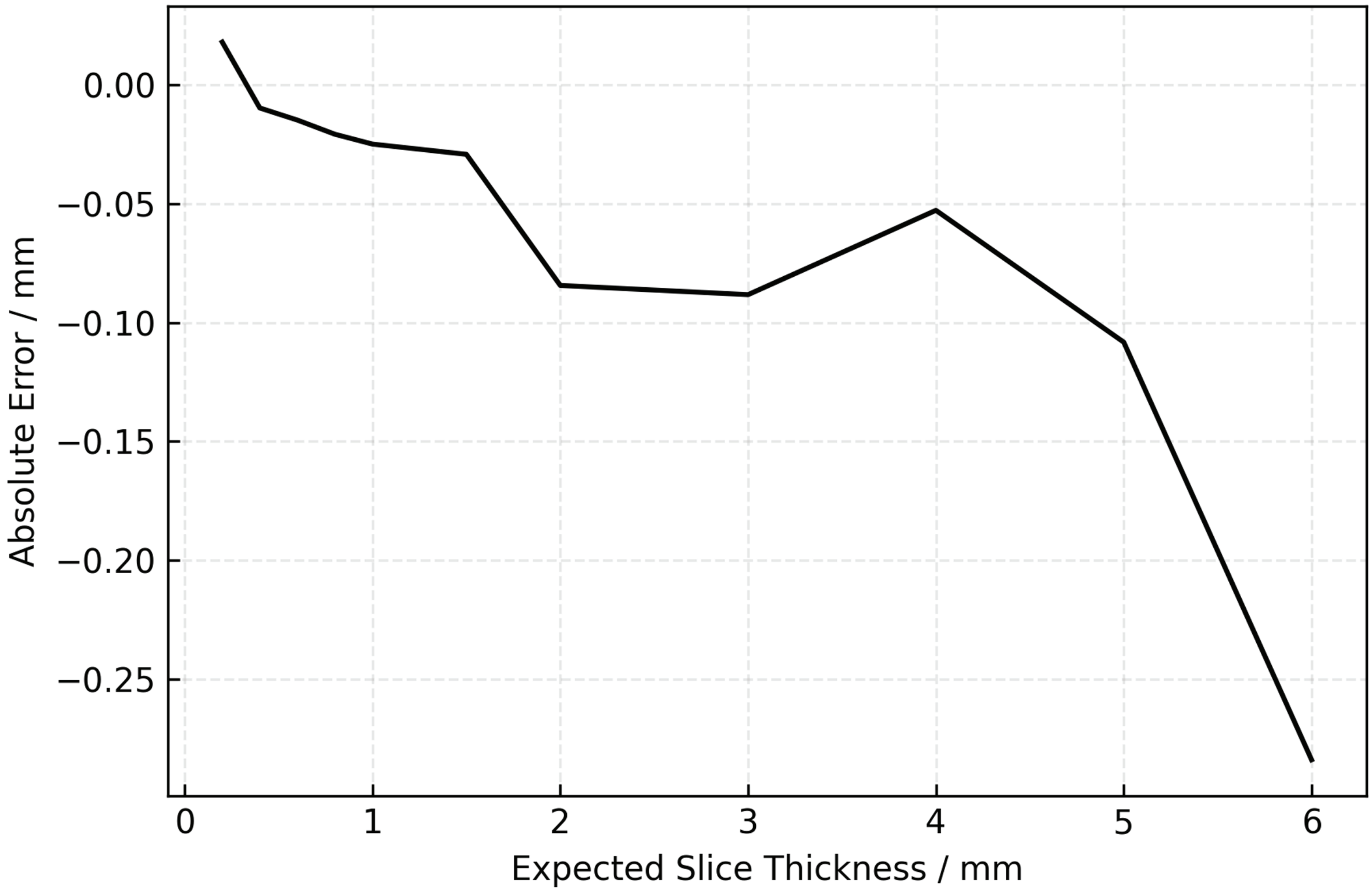

Figure S5: The absolute error from the expected slice thickness as measured for the protocols in Table S3.

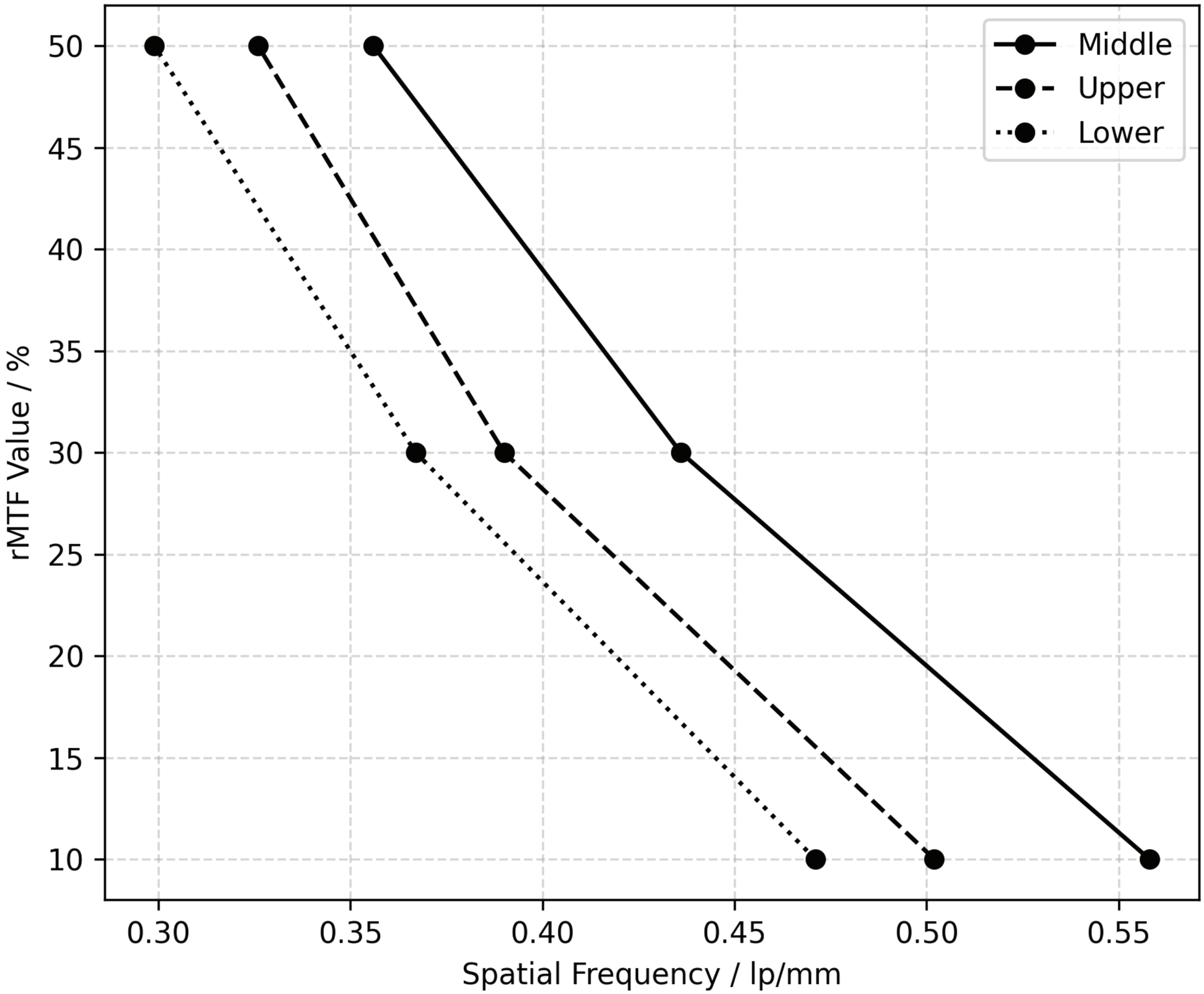

Figure S6: Relative MTF change as a function of CATPHAN position relative to isocenter. Middle is positioned at isocenter. The phantom center is positioned 11 cm above isocenter in the Upper position and 8 cm below isocenter is the Lower position. The data shown was acquired using a Br44 filter.

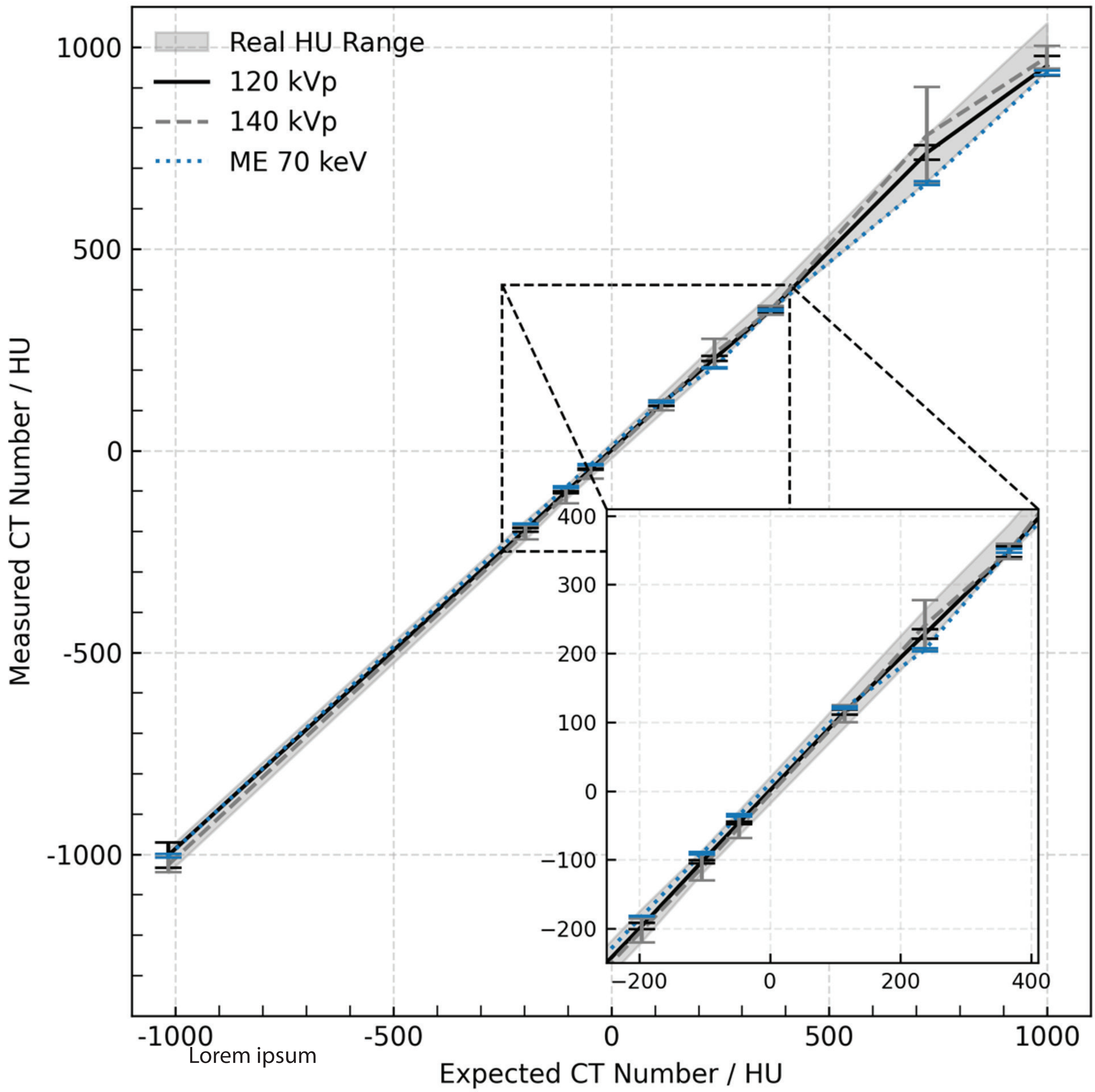

Figure S7: Mean CT number accuracy for CT protocols in Table S2 for the CATPHAN 604. The expected CT number values are shown as the shaded region in gray. Mean CT number and standard deviation between all protocols with peak voltage values of 120 kVp and 140 kVp as well as 70 keV monoenergetic scans are shown as separate lines.
