# Supplementary material for: Commissioning a single photon counting CT simulator for radiotherapy planning: Methods, challenges, and initial results
Source: J Appl Clin Med Phys. 2026 Sep 14;27(9):e70798. doi: 10.1002/acm2.70798 (PMC13575489; doi:10.1002/acm2.70798)
Supplement: Supplementary file 2 [file ACM2-27-e70798-s002.docx]

**Table S1: Electromechanical testing descriptions**

| **Parameter** | **Description** | **Tolerance** |
| --- | --- | --- |
| Gantry laser alignment and orthogonality with imaging isocenter | Align the Wilke phantom with the gantry lasers and image using the finest resolution (0.2 mm slice thickness and 0.11 mm in-plane resolution). Quantify how far the marks are from image isocenter. | ±2 mm |
| Gantry laser orthogonality over the projection range | With the Wilke phantom aligned to the gantry laser isocenter, drive the couch vertically and longitudinally and quantify the amount of laser drift from the Wilke markers. | ±2 mm over the entire projection length of the laser |
| Wall laser alignment and orthogonality with imaging isocenter and gantry lasers | Align the Wilke phantom with the wall laser isocenter. Drive the phantom into the bore by the offset distance of the wall lasers. Check alignment with gantry lasers. Image using the finest resolution (0.2 mm slice thickness and 0.11 mm in-plane resolution) and quantify how far the indentations are from image isocenter. | ±2 mm |
| Wall laser orthogonality over the projection range | With the Wilke phantom aligned to the wall laser isocenter, drive the couch vertically and longitudinally and quantify the amount of laser drift from the Wilke markers. | ±2 mm over the entire projection length of the laser |
| Couch vertical and longitudinal motion | Verify table is level.  Place 2 mm BB stickers at 400 mm increments longitudinally along the bottom of the curved couch. Align each BB at with the axial laser and record the digital position. Move the couch to align with the next BB and record the position. Record the difference in position.  Place a 450 mm ruler on the couch in the vertical or longitudinal orientation. Align with the appropriate laser and drive the couch a specified amount. Record the actual distance travelled on the ruler. | ±1 mm over the full range of couch motion |
| Couch vertical and longitudinal motion under scanner control | Verify table is level.  For vertical verification scan the TG 142 OMNI phantom was isocenter aligned with the -5 cm holes in the vertical pegs. Image the phantom, then shift the couch 10 cm down and image. Record the start and end positions indicated by the scanner. Measure the distance from isocenter of the +5 cm holes on the second image. Compare the distance measured in the image with the difference between the indicated positions of the couch.    Longitudinally image the full couch with the 2mm BBs placed at 400 mm increments along the center of the couch. Record the slice position when the each BB appears largest. Compare the indicated distances with the known distance of 400 mm. | ±1 mm over the full range of couch motion |
| Orientation of the CT-scanner couch with respect to the imaging plane | Verify the table top is level.  Using the laser phantom, align with the gantry lasers at the head of the table and image. Repeat with the phantom at the foot of the table and reimage. Images should show the phantom in the same location and image isocenter should correspond to the appropriate location in the phantom.  The phantom should also have BBs/holes/ridges on the edges (toward the edge of the FOV in a single slice). Those indicators should appear in the same vertical position and the opposite horizontal positions with respect to the center of the phantom in a single image and the same indicator should appear in the same location in the head image and the foot image. | ±2 mm over the length and width of the couch |
| Scout localization accuracy | Perform both an AP and lateral scout of an object with known dimensions.  Define a known volume to scan in each scout. Perform the scan. Check the start and ending positions of the scan as defined by the scanner and compare to the scan start and end positions defined by the scout.  Check that the size of the object as measured in each scout corresponds to the known size. | ±1 mm |
| Radiation profile width | Place a sheet of radiochromic film on the couch and align with isocenter.  Perform a single slice scan with each collimator setting and measure the FWHM on the film. | Manufacturer  specifications |
| Slice sensitivity | Scan the CATPHAN phantom with a bead source. Center the bead source at isocenter and image with the thinnest slices (0.2 mm) and least amount of slice spacing. Measure the maximum CT number in an ROI around the x,y position of the bead in each slice of the scan. Plot the max CT number vs. Slice position and find the FWHM of the peak. | ±1 mm from reference |
| **CQ Couch Test** |  |  |
| Scanner Table testing with load | Measure the deflection of the couch with and without load. | ±2 mm, +/- 2^o^ from reference |

| **Protocol Name** | **Scan Number** | **kVp** | **mAs** | **Pitch** | **Slice Thickness [mm]** | **Filter** |
| --- | --- | --- | --- | --- | --- | --- |
| Head and Neck | 1 | 140 | 239 | 0.35 | 0.4 | Hr44 |
| Head and Neck | 2 | 140 | 239 | 0.35 | 0.4 | Hr76 |
| Head and Neck | 3 | 140 | 239 | 0.35 | 0.4 | Qr40 (ME 70) |
| Thorax* | 4 | 140 | 14 | 1.0 | 0.4 | Br44 |
| Thorax* | 5 | 140 | 14 | 1.0 | 0.4 | Br76 |
| Thorax* | 6 | 140 | 14 | 1.0 | 0.4 | Bl64 |
| Thorax* | 7 | 140 | 14 | 1.0 | 0.4 | Qr44 (ME 70) |
| Non-Contrast Brain | 8 | 140 | 231 | 0.35 | 0.4 | Hr44 |
| Non-Contrast Brain | 9 | 140 | 231 | 0.35 | 0.4 | Hr76 |
| Non-Contrast Brain | 10 | 140 | 231 | 0.35 | 0.4 | Qr40 (ME 70) |
| Non-Contrast Brain | 11 | 140 | 233 | 0.35 | 0.4 | Hr44 |
| Non-Contrast Brain | 12 | 140 | 233 | 0.35 | 0.4 | Hr76 |
| Non-Contrast Brain | 13 | 140 | 233 | 0.35 | 0.4 | Qr40 (ME 70) |
| Forelimb | 14 | 140 | 41 | 0.8 | 0.4 | Br44 |
| Forelimb | 15 | 140 | 41 | 0.8 | 0.4 | Br76 |
| Forelimb | 16 | 140 | 41 | 0.8 | 0.4 | Qr40 (ME 70) |
| CTA Thorax | 17 | 140 | 9 | 1.5 | 0.4 | Br44 |
| CTA Thorax | 18 | 140 | 9 | 1.5 | 0.4 | Br76 |
| CTA Thorax | 19 | 140 | 9 | 1.5 | 0.4 | Qr40 (ME 70) |
| Abdomen | 20 | 140 | 10 | 0.8 | 0.4 | Br44 |
| Abdomen | 21 | 140 | 10 | 0.8 | 0.4 | Br76 |
| Abdomen | 22 | 140 | 10 | 0.8 | 0.4 | Qr40 (ME 70) |
| Trunk | 23 | 140 | 11 | 0.8 | 0.4 | Br44 |
| Trunk | 24 | 140 | 11 | 0.8 | 0.4 | Br76 |
| Trunk | 25 | 140 | 11 | 0.8 | 0.4 | Bl64 |
| Trunk | 26 | 140 | 11 | 0.8 | 0.4 | Qr40 (ME 70) |
| Trunk | 27 | 120 | 6 | 0.85 | 0.4 | Br44 |
| Trunk | 28 | 120 | 6 | 0.85 | 0.4 | Br96 |
| Trunk | 29 | 120 | 6 | 0.85 | 0.4 | Qr40 (ME 70) |
| Pelvis | 30 | 140 | 10 | 0.8 | 0.4 | Br44 |
| Pelvis | 31 | 140 | 10 | 0.8 | 0.4 | Br76 |
| Pelvis | 32 | 140 | 10 | 0.8 | 0.4 | Qr40 (ME 70) |
| Hindlimb | 33 | 140 | 51 | 0.8 | 0.4 | Br44 |
| Hindlimb | 34 | 140 | 51 | 0.8 | 0.4 | Br76 |
| Hindlimb | 35 | 140 | 51 | 0.8 | 0.4 | Qr40 (ME 70) |
| CTA Thorax | 36 | 140 | 9 | 1.5 | 0.4 | Br44 |
| CTA Thorax | 37 | 140 | 9 | 1.5 | 0.4 | Br64 |
| CTA Thorax | 38 | 140 | 9 | 1.5 | 0.4 | Qr40 (ME 70) |
| Venous | 39 | 140 | 9 | 1.5 | 0.4 | Br44 |
| Venous | 40 | 140 | 9 | 1.5 | 0.4 | Br64 |
| Venous | 41 | 140 | 9 | 1.5 | 0.4 | Qr40 (ME 70) |
| Delayed | 42 | 140 | 9 | 1.5 | 0.4 | Br44 |
| Delayed | 43 | 140 | 9 | 1.5 | 0.4 | Br64 |
| Delayed | 44 | 140 | 9 | 1.5 | 0.4 | Qr40 (ME 70) |
| CTA Coronary | 45 | 140 | 183 | 0.18 | 0.4 | Qr44 (Best Diastolic 82%) |
| CTA Coronary | 46 | 140 | 183 | 0.18 | 0.4 | Qr44 (Best Systolic 185ms) |

Table S2: CT protocols evaluated for baseline image quality. ME 70 indicates monoenergetic images of 70 keV. The protocols marked with asterisks (*) were evaluated for at various distances vertically from the image center as well.

| Protocol Name | Scan Number | kVp | mAs | Pitch | Slice Thickness [mm] | Filter |
| --- | --- | --- | --- | --- | --- | --- |
| Body | 1 | 140 | 88 | 0.85 | 0.2 | Br40 |
| Body | 2 | 140 | 88 | 0.85 | 0.4 | Br40 |
| Body | 3 | 140 | 88 | 0.85 | 0.6 | Br40 |
| Body | 4 | 140 | 88 | 0.85 | 0.8 | Br40 |
| Body | 5 | 140 | 88 | 0.85 | 1.0 | Br40 |
| Body | 6 | 140 | 88 | 0.85 | 1.5 | Br40 |
| Body | 7 | 140 | 88 | 0.85 | 2.0 | Br40 |
| Body | 8 | 140 | 88 | 0.85 | 3.0 | Br40 |
| Body | 9 | 140 | 88 | 0.85 | 4.0 | Br40 |
| Body | 10 | 140 | 88 | 0.85 | 5.0 | Br40 |
| Body | 11 | 140 | 88 | 0.85 | 6.0 | Br40 |

Table S3: CT protocols evaluated for the effect of slice thickness on image quality.

| Patient Size | CTDI phantom | Scan Mode / Name |  | Tube Voltage | Current | Effective | Scan Time | Measured CTDI_vol | Nomimal reported CTDI_vol | Discrepancy | Measured DLP | Nominal DLP | Discrepancy |
| --- | --- | --- | --- | --- | --- | --- | --- | --- | --- | --- | --- | --- | --- |
|  |  |  | Shaped filter | kVp | mA | mAs | s | mGy | mGy | % | mGy cm | mGy . cm | % |
| Adult | BODY | RoutineSequenceAdultAbdomenQuantumPlus | Standard | 140 | 158 | 79 | 0.5 | 10.9 | 10.3 | 5.4% | 52.3 | 52.3 | 0.0% |
| Adult | BODY | RoutineSequenceAdultAbdomenQuantum | Standard | 90 | 542 | 271 | 0.5 | 12.0 | 11.0 | 9.0% | 57.6 | 55.7 | 3.3% |
| Child | BODY | RoutineSequenceChildAbdomenQuantumPlus | Narrow | 140 | 80 | 40 | 0.5 | 4.5 | 4.2 | 7.4% | 21.6 | 21.8 | -0.9% |
| Child | BODY | RoutineSequenceChildAbdomenQuantum | Narrow | 90 | 270 | 136 | 0.5 | 4.7 | 4.3 | 10.1% | 22.7 | 21.4 | 5.9% |
| Adult | HEAD | RoutineSequenceAdultHeadQuantumPlus | Standard | 120 | 234 | 117 | 0.5 | 19.7 | 19.8 | -0.7% | 113.3 | 114.0 | -0.6% |
| Adult | HEAD | RoutineSequenceAdultHeadQuantum | Standard | 90 | 506 | 253 | 0.5 | 20.6 | 20.6 | 0.1% | 118.8 | 119.0 | -0.2% |
| Child | HEAD | RoutineSequenceChildHeadQuantum | Standard | 90 | 252 | 126 | 0.5 | 10.3 | 10.3 | -0.2% | 59.2 | 59.1 | 0.1% |
| Child | HEAD | RoutineSequenceChildHeadQuantumPlus | Standard | 120 | 118 | 59 | 0.5 | 9.9 | 10.0 | -0.6% | 57.2 | 57.4 | -0.3% |

Table S4: Nominal versus measured CTDI_vol and DLP values from the Siemens Naeotom Alpha CT Scanner

| Protocol Name | Scan Number | kVp | mAs | Pitch | Slice Thickness [mm] | Filter |
| --- | --- | --- | --- | --- | --- | --- |
| Head and Neck+ | 1 | 120 | 218 | 0.35 | 0.4 | Hr44 |
| Head and Neck | 2 | 120 | 218 | 0.35 | 0.4 | Hr76 |
| Head and Neck | 3 | 120 | 218 | 0.35 | 0.4 | Qr44 |
| Head and Neck* | 4 | 120 | 218 | 0.35 | 1.0 | Hr44 |
| Head and Neck | 5 | 120 | 218 | 0.35 | 1.0 | Hr76 |
| Head and Neck* | 6 | 120 | 218 | 0.35 | 2.0 | Hr44 |
| Head and Neck | 7 | 120 | 218 | 0.35 | 2.0 | Hr76 |
| Head and Neck+ | 8 | 140 | 159 | 0.35 | 0.4 | Hr44 |
| Head and Neck | 9 | 140 | 83 | 0.80 | 0.4 | Hr44 |
| Head and Neck* | 10 | 140 | 159 | 0.35 | 0.4 | Hr76 |
| Head and Neck | 11 | 140 | 159 | 0.35 | 1.0 | Hr44 |
| Head and Neck | 12 | 140 | 159 | 0.35 | 2.0 | Hr44 |
| Head and Neck* | 13 | 140 | 159 | 0.35 | 2.0 | Hr76 |
| Trunk* | 14 | 140 | 90 | 0.80 | 0.4 | Br44 |
| Trunk* | 15 | 140 | 90 | 0.80 | 0.4 | Bl64 |
| Abdomen | 16 | 140 | 90 | 0.80 | 0.4 | Br44 |
| Abdomen | 17 | 140 | 90 | 0.80 | 0.4 | Br76 |
| Pelvis* | 18 | 140 | 88 | 0.80 | 0.4 | Br44 |
| Pelvis | 19 | 140 | 83 | 0.80 | 0.4 | Br76 |
| Lower Limb | 20 | 140 | 88 | 0.80 | 0.4 | Br76 |
| Lower Limb | 21 | 140 | 159 | 0.35 | 0.4 | Br44 |
| Upper Limb* | 22 | 140 | 88 | 0.80 | 0.4 | Br44 |
| Upper Limb | 23 | 140 | 127 | 1.00 | 0.4 | Br76 |
| Thorax | 24 | 140 | 127 | 1.00 | 0.4 | Br44 |
| Thorax* | 25 | 140 | 127 | 1.00 | 0.4 | Br76 |
| Thorax | 26 | 140 | 159 | 0.35 | 0.4 | Bl64 |

Table S5: CT protocols evaluated for relative electron density measurements. Protocols with hatch (+) identify the reference scan for assessing differences among the protocols. Protocols with asterisks (*) are those protocols whose RED measured data are statistically different from baselines.
